# Synthesis of Polymer-Based Magnetic Nanocomposite for Multi-Pollutants Removal from Water

**DOI:** 10.3390/polym13111742

**Published:** 2021-05-26

**Authors:** Fatimah Mohammed Alzahrani, Norah Salem Alsaiari, Khadijah Mohammedsaleh Katubi, Abdelfattah Amari, Faouzi Ben Rebah, Mohamed A. Tahoon

**Affiliations:** 1 Chemistry Department, College of Science, Princess Nourah bint Abdulrahman University, Riyadh 11671, Saudi Arabia; fmalzahrani@pnu.edu.sa; 2 Department of Chemical Engineering, College of Engineering, King Khalid University, Abha 61411, Saudi Arabia; 3 Research Laboratory, Department of Chemical Engineering, Energy and Environment, National School of Engineers, Gabes University, Gabes 6072, Tunisia; 4 Department of Chemistry, College of Science, King Khalid University, Abha 61413, Saudi Arabia; tahooon_87@yahoo.com; 5 Higher Institute of Biotechnology of Sfax (ISBS), Sfax University, Sfax 3000, Tunisia; 6 Chemistry Department, Faculty of Science, Mansoura University, Mansoura 35516, Egypt

**Keywords:** polymers, magnetic nanomaterials, adsorption, Congo red removal, chromium removal

## Abstract

A magnetic polymer-based nanocomposite was fabricated by the modification of an Fe_3_O_4_/SiO_2_ magnetic composite with polypyrrole (PPy) via co-precipitation polymerization to form PPy/Fe_3_O_4_/SiO_2_ for the removal of Congo red dye (CR) and hexavalent chromium Cr(VI) ions from water. The nanocomposite was characterized using various techniques including X-ray diffraction (XRD), Fourier transform infrared spectroscopy (FTIR), scanning electron microscope (SEM), vibration sample magnetometer, and thermogravimetric analysis (TGA). The results confirm the successful fabrication of the nanocomposite in the size of nanometers. The effect of different conditions such as the contact time, adsorbent dosage, solution pH, and initial concentration on the adsorption process was investigated. The adsorption isotherm suggested monolayer adsorption of both contaminants over the PPy/Fe_3_O_4_/SiO_2_ nanocomposite following a Langmuir isotherm, with maximum adsorption of 361 and 298 mg.g^−1^ for CR dye and Cr(VI), respectively. Furthermore, the effect of water type on the adsorption process was examined, indicating the applicability of the PPy/Fe_3_O_4_/SiO_2_ nanocomposite for real sample treatment. Interestingly, the reusability of the nanocomposite for the removal of the studied contaminants was investigated with good results even after six successive cycles. All results make this nanocomposite a promising material for water treatment.

## 1. Introduction

Industrial wastewater treatment has become increasingly complex in recent decades as a result of the rapid industrialization and the presence of complex mixtures of toxic metal ions and organic dyes that harm human health and the environment [1,2,3]. The textile, paper, and plastic industries are the most common manufacturing purposes including dyes as important aromatic compounds [4]. In addition to their carcinogenic effects, contaminated drinking water with dyes causes various symptoms including severe headaches, skin irritation, and acute diarrhea [5]. Similarly, metal ions are not less dangerous than organic dyes due to their high toxicity when discharged in water supplies [6]. One of these toxic metals, hexavalent chromium (Cr(VI)), is classified as a very toxic and carcinogenic metal, causing nephritis, gastrointestinal ulceration, and cancer in the digestive tract [7]. The accepted concentrations of hexavalent chromium in drinking water and industrial wastewater are 5.0 and 200.0 μg/L, respectively [8]. Therefore, the removal of such pollutants from water has become a challenge for environmental engineers. Several techniques have been applied for the removal of these harmful metallic ions and dyes from water and wastewater such as membrane filtration [9], photocatalytic reduction [10], biological treatment [11], precipitation [12], electrocoagulation [13], and adsorption [14,15].

The adsorption technique is considered as the most effective treatment method due to several reasons such as its high removal efficiency, economic viability, and simple processing, which make it a cost-effective method for pollutant removal from water [16,17,18]. Recently, nanomaterials with exceptional properties [19,20,21,22] have been found to be useful in different fields, especially water treatment. Magnetic nanoparticles as a class of nanomaterials have been widely used in different fields, due to their ease of separation using an external magnet, high specific surface area, and simple modification. Generally, these magnetic nanomaterials include elements with magnetic properties in their chemical structure such as iron, nickel, and cobalt. The exceptional properties of iron oxide (Fe_3_O_4_) nanoparticles such as high adsorption, superparamagnetic behavior, good compatibility, low toxicity, high surface energy, and large surface area have attracted attention in recent years. Thus, these nanoparticles are appropriate for the elimination of targeted molecules. The toxicity and safety of using nanoparticles in water and food applications are important points. Interestingly, the non-toxicity of Fe_3_O_4_ allowed its wide applications in water and food fields among all other magnetic nanoparticles [23]. To increase the adsorption capacity and efficiency of Fe_3_O_4_ nanoparticles, they are usually modified by other organic or inorganic compounds to save the functional groups for capturing the target molecules. The surface of Fe_3_O_4_ magnetic nanoparticles can be easily modified using silica (silicon oxide) that has a high surface/volume ratio and, subsequently, can improve the adsorption capacity of Fe_3_O_4_ nanoparticles. Silica is widely used in different industries such as ceramics, ferrosilicon production, porcelain, and glassmaking, and as semiconductors in electronics [24]. This makes silica a promising material for different applications. The structural characteristics of silicon oxide allow carrying macromolecules such as proteins and polymers that associate the potential properties of polymers and the silica mechanical stability. This association of different properties is paving the way to numerous technological uses.

Among various polymers, polypyrrole (PPy) has received considerable interest due to its biocompatibility, low cost, excellent chemical stability, and conductivity. Moreover, PPy can be easily synthesized and used in different applications such as the nanomedicine sector, electrochemical sensors, and water treatment. In this context, the use of PPy to adsorb pollutants such as heavy metals has been reported [25,26,27]. Thus, PPy is an excellent choice for the modification of magnetic composites, especially the PPy polymer previously composited with magnetic nanoparticles for different applications such as lithium-ion batteries [28], due to its good compatibility with different nanoparticles.

Therefore, we can conclude that the use of composite materials for water treatment purposes can enhance the chelation and adsorption power of the materials toward targeted molecules. Herein, we synthesized an Fe_3_O_4_/SiO_2_/PPy magnetic nanocomposite for water treatment purposes. In the first part of the paper, we report the synthesis and characterization of the magnetic nanocomposite. In the second part of the paper, we report the application of the synthesized materials to the removal of Cr(VI) and Congo red dye with a discussion of the compositing process’s role in the improvement of the material removal efficiency.

## 2. Materials and Methods

### 2.1. Chemicals

Congo red (97%) and K_2_Cr_2_O_7_ (99.9%) were supplied by Sigma-Aldrich (Cairo, Egypt), while the monomer of pyrrole (98.0%) was purchased from Merck Co. (Cairo, Egypt). Tetraethyl orthosilicate (TEOS, 98%), aqueous ammonia (25%), ammonium ferrous sulfate salts (99%), and ferric chloride (98%) were supplied by Sigma-Aldrich (Cairo, Egypt). Hydrochloric acid (35.0%) and sodium hydroxide (97.0%) for the pH adjustment were supplied by El Nasr Co. (Cairo, Egypt).

### 2.2. Synthesis of Fe_3_O_4_/SiO_2_/PPy Magnetic Nanocomposite

The synthesis of the Fe_3_O_4_/SiO_2_/PPy magnetic nanocomposite was achieved in three steps. The first step included the synthesis of Fe_3_O_4_ nanoparticles, which was followed by the second step consisting of the modification of Fe_3_O_4_ nanoparticles with SiO_2_. The third step included the formation of polypyrrole over the Fe_3_O_4_/SiO_2_ composite. For Fe_3_O_4_ nanoparticle synthesis, we followed the literature [29] in which hydrochloric acid (HCl, 0.5 M, 100 mL) was used for dissolving ammonia iron sulfate ((NH_4_)_2_Fe(SO_4_)_2_·6H_2_O), 7.85g) and ferric chloride (FeCl_3_, 10.82 g), followed by placing this mixture at 30 °C in an ultrasound bath for half an hour. After that, the temperature of the mixture was adjusted at 80 °C, aqueous ammonia (25%, 60 mL) was added drop-wise, and the solution was stirred at 1500 rpm to form the Fe_3_O_4_ nanoparticles as a black precipitate. The stirring process of the mixture was continued up to one hour followed by collecting the nanoparticles via an external magnet, and the collected Fe_3_O_4_ nanoparticles were washed several times with distilled water and dried for half a day at 70 °C in an oven. Finally, Fe_3_O_4_ nanoparticles were ready to be used. In the second step, Fe_3_O_4_ nanoparticles were modified with SiO_2_ by using tetraethyl orthosilicate (TEOS). This was achieved by using a mixture of distilled H_2_O (40 mL) and ethyl alcohol (99.9%, 170 mL) to dissolve 2.0 g of synthesized Fe_3_O_4_ nanoparticles. Then, after adding 6 mL of aqueous ammonia, this mixture was placed for 15.0 min at 30 °C in an ultrasound bath. After the complete dispersion of Fe_3_O_4_ with vigorous stirring up to half a day under argon gas environment, 2 mL of TEOS was added to the solution. The synthesized Fe_3_O_4_/SiO_2_ was then collected by an external magnet, washed several times with absolute ethyl alcohol, and finally dried at 70 °C in an oven for four hours. The final step was the formation of PPy over the synthesized Fe_3_O_4_/SiO_2_ composite via the co-precipitation chemical method. In a glass beaker, 100 mL of distilled water was used to disperse 1.0 g of Fe_3_O_4_/SiO_2_ nanoparticles. For the complete dispersion of nanoparticles, the beaker was placed for 15.0 min at 30 °C in an ultrasound bath. After that, the solution was stirred vigorously and during that, pyrrole (0.1 M) was added and the stirring was continued up to two hours followed by the addition of ferric chloride (0.1 M, 50.0 mL); the reaction was continued for an additional three hours. Finally, the synthesized nanocomposite of PPy/Fe_3_O_4_/SiO_2_ was collected by an external magnet, washed several times with absolute ethyl alcohol, and then dried in an oven at 60.0 °C for 6.0 h.

### 2.3. Material Characterization

The synthesized materials were characterized using various techniques including X-ray diffraction (XRD), magnetometer, Fourier transform infrared spectroscopy (FT-IR), scanning electron microscope (SEM), and thermogravimetric analysis (TGA) measurement. A GNR APD-2000 PRO (GNR, Cairo, Egypt) diffractometer was used to measure XRD using Cu Ka radiation (λ = 1.5406 Å), operating at 45 kV. The diffraction intensities were recorded over 2θ ranging from 5° to 60° with a constant scanning rate of 1° min^−1^. A vibrating sample magnetometer (Lake Shore 7410, Lake Shore (Cryotronics Inc., Westerville, OH, USA) was used to measure the magnetization of the nanocomposite. A Bruker, Tensor 27 FT–IR (BRUKER, Karlsruhe, Germany) spectrophotometer was used to perform FT-IR spectra in the range of 400–4000 cm^−1^ at room temperature and collected these at a resolution of 4 cm^−1^. An SEM Hitachi S4800 (Hitachi, Tokyo, Japan) was used to study the morphology of the synthesized material. A Perkin Elmer STA 6000 (PerkinElmer Inc., Shelton, USA) was used to measure the thermogravimetric analysis for the evaluation of thermal stability.

### 2.4. Adsorption Studies

#### 2.4.1. The Effect of Contact Time

The effect of the contact time on the removal of Cr(VI) and Congo red from water using the PPy/Fe_3_O_4_/SiO_2_ nanocomposite was studied using a volume of 100 mL of contaminated water (100 mg.L^−1^) and adsorbent mass of 0.02 g at pH of 4.0. The solution shaking was conducted up to 24 h with sampling at various times to investigate the remaining contaminants using ICP and a UV–Vis spectrophotometer. The adsorbent was collected after each experiment using a magnet. All experiments were performed in triplicate.

#### 2.4.2. The Effect of Adsorbent Dosage

Different masses of the adsorbent PPy/Fe_3_O_4_/SiO_2_ nanocomposite were used to study the effect of adsorbent dosage on the removal of Cr(VI) and Congo red. The definite mass of adsorbent was mixed with 100.0 mL of polluted water with pH of 4.0 up to 12 h. When equilibrium was reached, the adsorbent was collected with a magnet, and the solution was examined for the presence of Cr(VI) and Congo red. Experiments were performed in triplicate.

#### 2.4.3. The Effect of Solution pH

A pH range of 3.0 to 7.0 was used to investigate the pH effect on the removal of Cr(VI) and Congo red using the PPy/Fe_3_O_4_/SiO_2_ nanocomposite. The adsorbent mass used was 0.05 g, the time was 12.0 h, and the solution volume was 100.0 mL. After each experiment, magnetic separation was used to collect the adsorbent, and the solution was examined for any residual pollutants using ICP and a UV–Vis spectrophotometer (Hach, CO, USA). Experiments were performed in triplicate.

#### 2.4.4. The Adsorption Isotherm

To study the maximum adsorption capacity and the type of the adsorption process of Cr(VI) and Congo red over the synthesized PPy/Fe_3_O_4_/SiO_2_ nanocomposite, the contaminated solution (100.0 mL) was mixed with 0.02 g of the composite for 24.0 h at pH 4.0. The adsorption mechanism was represented by three isotherm models: Freundlich, Langmuir, and Temkin.

#### 2.4.5. The Regeneration Study

To assess the commercial application of an adsorbent, it is necessary to determine the reusability of the material for the effective removal of contaminants several times. This was achieved by performing the adsorption experiment 6.0 successive times by mixing the contaminated water (100 mg.L^−1^, 100 mL, pH 4.0) with adsorbent (0.05 g) for 12.0 h. After each cycle, the adsorbent was collected with an external magnet, washed with distilled water extensively, and then dried at 50.0 °C for 5.0 h to be used in the next cycle. Experiments were performed in triplicate.

#### 2.4.6. Water Type Effect

Different types of water including wastewater, groundwater, tap water, and distilled water were used to determine the water type effect on the removal of Cr(VI) and Congo red using the synthesized PPy/Fe_3_O_4_/SiO_2_ nanocomposite. Polluted solution prepared using target water with a concentration of 100.0 mg.L^−1^ and volume of 100.0 mL was mixed with 0.05 g of adsorbent for 12 h. After each experiment, the solution was examined for the presence of contaminants.

## 3. Results and Discussion

### 3.1. Nanocomposite Characterization

**Figure 1:**
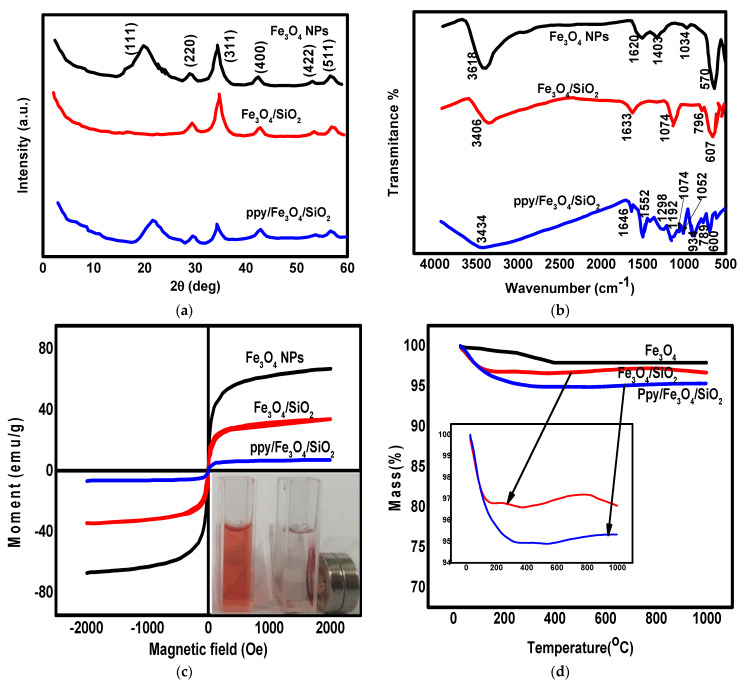
XRD (**a**), FT-IR (**b**), magnetization curves (**c**) (inset: separation of magnetic nanocomposite after adsorption with an external magnet), and thermogravimetric analysis (TGA) (**d**) of synthesized Fe_3_O_4_ nanoparticles, Fe_3_O_4_/SiO_2_ nanocomposite, and PPy/Fe_3_O_4_/SiO_2_ nanocomposite.

The synthesized materials including Fe_3_O_4_, Fe_3_O_4_/SiO_2_, and PPy/Fe_3_O_4_/SiO_2_ nanomaterials were investigated using different techniques such as X-ray diffraction (XRD), Fourier transform infrared spectroscopy (FT-IR), and scanning electron microscope (SEM). The XRD spectra of Fe_3_O_4_ nanoparticles, Fe_3_O_4_/SiO_2_, and PPy/Fe_3_O_4_/SiO_2_ are shown in Figure 1a. The XRD of Fe_3_O_4_ nanoparticles alone indicated the cubic phase structure, and the peaks correspond to the (511), (422), (400), (311), and (220) crystalline planes [30]. The XRD of the Fe_3_O_4_/SiO_2_ composite showed the appearance of all Fe_3_O_4_ peaks with the reduction in the peak intensity and the disappearance of the peak at 2θ = 20 [31] indicating the crystallinity decrease in Fe_3_O_4_ by the combination with amorphous SiO_2_. The XRD of the PPy/Fe_3_O_4_/SiO_2_ nanocomposite showed the appearance of all Fe_3_O_4_ peaks, and there was an overlap between the two peaks of Fe_3_O_4_ and PPy at 2θ = 20 that made this peak wider [32]. The diffraction intensity of this peak showed a clearly increase linked to the marked increase in the thickness of the polypyrrole shell, indicating the existence of amorphous polypyrrole in the sample. Additionally, the amorphous structure of SiO_2_ and PPy caused a reduction in the intensity of Fe_3_O_4_ peaks. This indicates the formation of the composite PPy/Fe_3_O_4_/SiO_2_. The XRD results are in agreement with previous studies of Fe_3_O_4_, Fe_3_O_4_/SiO_2_, and PPy [31,32,33]. For the investigation of the functional groups present, the FT-IR spectra of Fe_3_O_4_ nanoparticles, Fe_3_O_4_/SiO_2_, and PPy/Fe_3_O_4_/SiO_2_ are shown in Figure 1b. The FT-IR spectrum of Fe_3_O_4_ nanoparticles showed the appearance of an Fe-O band at 570 cm^−1^ with the appearance of a hydroxyl group band at 3418 cm^−1^, due to the existence of H_2_O in the nanoparticle structure [34]. In the spectra of Fe_3_O_4_/SiO_2_, the peaks appeared at 796 cm^−1^ and 1074 cm^−1^ and correspond to an Fe-O-Si bond and a Si-O bond, respectively. Additionally, the same peaks of Fe_3_O_4_ nanoparticles corresponding to hydroxyl group and Fe-O are observed with a small shift due to the interaction between Fe_3_O_4_ and SiO_2_, while the FT-IR spectrum of the nanocomposite PPy/Fe_3_O_4_/SiO_2_ showed the existence of all peaks corresponding to Si-O-Fe, Si-O, and Fe-O with a small shift due to the interaction between Fe_3_O_4_ and PPy [35]. Additionally, the NOH peak appeared at 934 cm^−1^ and the C-N peak appeared at 1192 cm^−1^. Additionally, the C-H vibrations were represented by the peaks at 1052, 1300, and 1298 cm^−1^ [36]. The pyrrole ring vibrations were represented by the peaks at 1552 and 1547 cm^−1^. The wide peak observed at the range of 3500–3000 is characteristic of PPy composites, due to the N-H bonds’ large number [28]. Furthermore, the N-H and O-H vibrations were represented by the peaks at 3447 and 3434 cm^−1^ [28,36]. The results of FT-IR are in excellent agreement with previous studies of Fe_3_O_4_, Fe_3_O_4_/SiO_2_ [29], and PPy and its compounds [37].

An advantage of the present nanocomposite is its magnetic properties that allow its separation from the experimental media. Therefore, the magnetization curves of the synthesized materials Fe_3_O_4_, Fe_3_O_4_/SiO_2_, and PPy/Fe_3_O_4_/SiO_2_ at room temperature are shown in Figure 1c. According to Figure 1c, the highest saturation magnetization was observed at 68 emu.g^−1^ for Fe_3_O_4_ nanoparticles. This value dropped to 35 emu.g^−1^ after the modification of Fe_3_O_4_ with silica, indicating the effective interaction between Fe_3_O_4_ and SiO_2_ [38]. Moreover, the successful modification of the composite Fe_3_O_4_/SiO_2_ with PPy was indicated by the dropped value of saturation magnetization to 8 emu.g^−1^, indicating that the SiO_2_ layer was thinner than the PPy layer [39]. It can be noticed that all synthesized materials have good magnetic properties, allowing magnetic separation using an external magnet, as shown in (Figure 1c, inset).

The thermal stability of the synthesized nanomaterials (Fe_3_O_4_, Fe_3_O_4_/SiO_2_, and PPy/Fe_3_O_4_/SiO_2_) was investigated using thermogravimetric analysis (TGA), as shown in Figure 1d. According to the TGA curve of Fe_3_O_4_, there is a minor weight loss (in the temperature range of room temperature to 10,000 °C) equal to about 3.0% that is attributed to the evaporation of the chemical or physical attached water or hydroxyl groups on the surface of Fe_3_O_4_ nanoparticles. The TGA curve of Fe_3_O_4_/SiO_2_ indicated the successful deposition of thermally stable SiO_2_ on the Fe_3_O_4_ surface as there was only 3.5% weight loss in the temperature from room temperature to 161 °C, which is attributed to the evaporation of adsorbed water. The TGA curve of the PPy/Fe_3_O_4_/SiO_2_ nanocomposite showed 5.0% weight loss at the temperature range from room temperature to 300.0 °C, which is attributed to the removal of the PPy monomer and the evaporation of adsorbed water. Ppy chains have good thermal stability and can be destroyed at high temperatures only. This fact is indicated by the sharp peak at 640.0 °C and weak peak at 400.0 to 500.0 °C. The low thermal stability of the PPy layer resulted in higher weight loss of PPy/Fe_3_O_4_/SiO_2_ compared to Fe_3_O_4_/SiO_2_. The TGA results indicate the good incorporation of PPy and silica in the synthesized PPy/Fe_3_O_4_/SiO_2_ nanocomposite.

**Figure 2:**
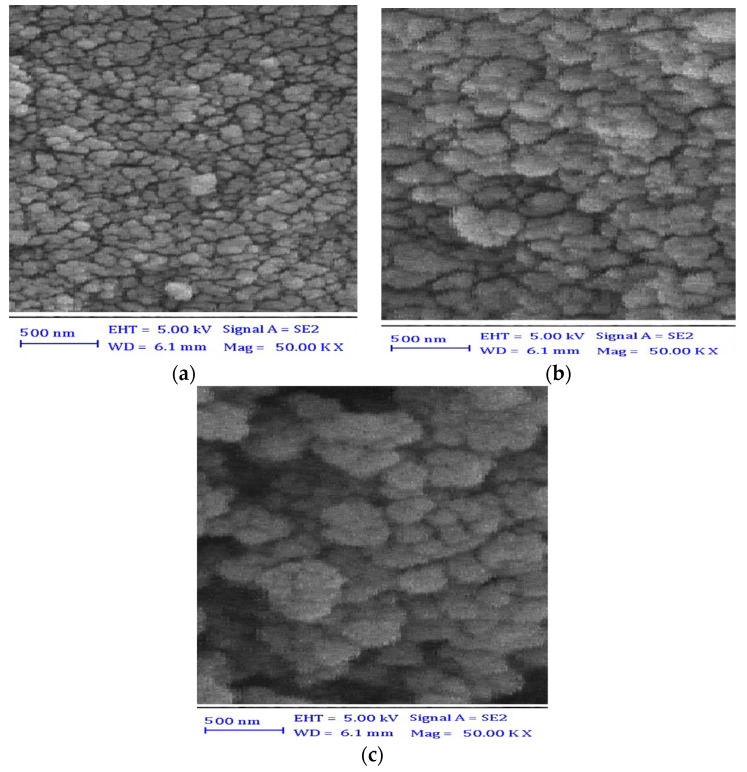
SEM images of Fe_3_O_4_ nanoparticles (**a**), Fe_3_O_4_/SiO_2_ nanocomposite (**b**), and PPy/Fe_3_O_4_/SiO_2_ nanocomposite (**c**).

For determining the surface structure and morphology of the synthesized materials, the SEM images were assessed (Figure 2).

The SEM image of Fe_3_O_4_ nanoparticles indicated their granular shape with a size range of 40.0 to 100.0 nm (in Figure 1a). Similar granular shapes with no big differences in porosity, morphology, and structure were observed between the Fe_3_O_4_/SiO_2_ composite (Figure 2b) and Fe_3_O_4_ nanoparticles. However, the morphology of the particles was changed by the addition of PPy, as shown in Figure 2c. The particles became cabbage in shape with an increase in their size as expected due to the deposition of pyrrole over Fe_3_O_4_ firstly, followed by the synthesis of PPy, causing the size increase. The present results are in agreement with the previous studies of Fe_3_O_4_ nanoparticles, Fe_3_O_4_/SiO_2_ composites [29], and PPy composites [40] from the view of particle shape, morphology, and porosity.

### 3.2. Adsorption Studies

#### 3.2.1. Effect of Contact Time

**Figure 3:**
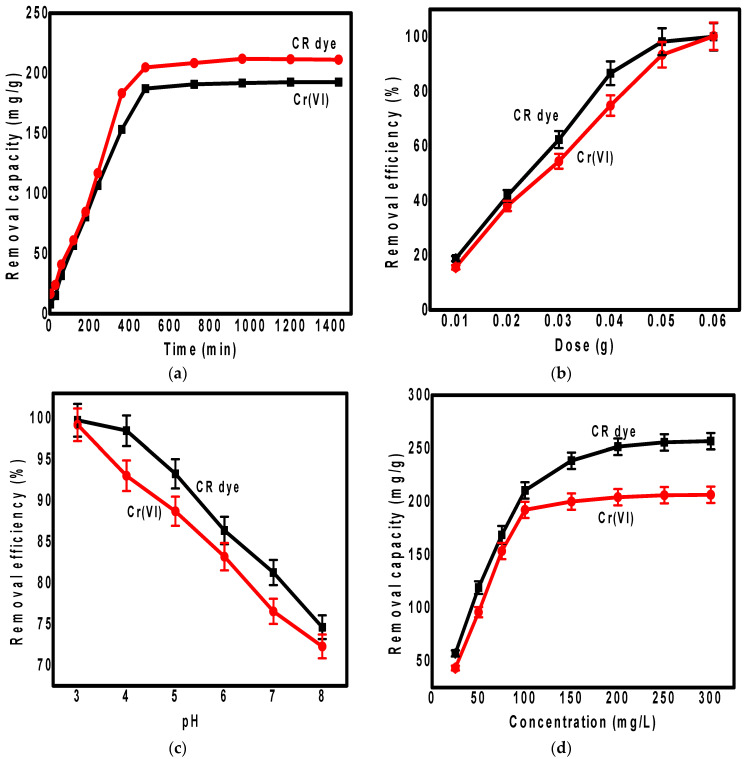
The contact time effect (**a**), adsorbent dosage effect (**b**), pH effect (**c**), and initial concentration effect (**d**) on the removal of Congo red dye and Cr(VI) from water using PPy/Fe_3_O_4_/SiO_2_ nanocomposite.

The effect of the contact time on the removal of Cr(VI) and Congo red dye from water using the magnetic PPy/Fe_3_O_4_/SiO_2_ nanocomposite was studied, as shown in Figure 3a. According to Figure 3a, there were two stages of removal of both pollutants. The first stage showed a rapid removal of Cr(VI) and Congo red dye that appeared in the plot as a linear curve. The second stage showed stable removal of Cr(VI) and Congo red dye, which appeared in the plot as a plateau curve, meaning that the adsorption process reached equilibrium.

This behavior can be explained in that the first stage occurred due to the existence of a large number of empty adsorption sites on the adsorbent surface, allowing the rapid capture of pollutants within a short period. After that, the adsorption sites became filled, and equilibrium was achieved [41,42]. According to Figure 3a, the Cr(VI) and Congo red dye removal by the magnetic PPy/Fe_3_O_4_/SiO_2_ nanocomposite increased from 8.0 to 193.0 mg.g^−1^ and from 17.0 to 213.0 mg.g^−1^, respectively, when the contact time increased from 5.0 to 1440.0 min. As clearly shown in the contact time plot, 480.0 min was the point at which equilibrium was reached for the removal of Cr(VI) and Congo red dye using the PPy/Fe_3_O_4_/SiO_2_ nanocomposite.

#### 3.2.2. Effect of Adsorbent Dosage

Figure 3b shows the effect of adsorbent dosages on the removal of Cr(VI) and Congo red dye from water using the PPy/Fe_3_O_4_/SiO_2_ nanocomposite. This was achieved using different masses of the nanocomposite as follows: 0.06, 0.05, 0.04, 0.03, 0.02, and 0.01 g. According to Figure 3b, the increased dose of the nanocomposite led to an increase in the removal percentage of both pollutants. This behavior is attributed to the existence of accessible and sufficient adsorption sites for the uptake of Cr(VI) and Congo red, which are increased by adding more quantities of the adsorbent, as reported in previous studies [43]. According to the Figure 3b, the removal efficiencies of Cr(VI) and Congo red were increased from 16.0% to 98.0% and from 19.0% to 99.5%, respectively, when the mass of the PPy/Fe_3_O_4_/SiO_2_ nanocomposite increased from 0.01 to 0.06 g. When the adsorbent mass increased from 0.05 to 0.06 g, there was no significant increase in the removal efficiencies. Therefore, 0.05 g was considered the optimum adsorbent dose for the removal of both contaminants using the PPy/Fe_3_O_4_/SiO_2_ nanocomposite. This behavior may be attributed to the saturation of the adsorption sites or the agglomeration of adsorbent particles when their quantity increased, leading to the constant removal efficiency [44].

#### 3.2.3. Effect of Solution pH

The pH effect on the removal of Cr(VI) and Congo red using the PPy/Fe_3_O_4_/SiO_2_ nanocomposite was investigated in the range of 3.0 to 8.0, as shown in Figure 3c. According to Figure 3c, the highest removal efficiencies were achieved at the lowest pH (3.0 to 4.0) with removal values of 99.0% and 99.7% for Cr(VI) and Congo red, respectively. Moreover, the pH increase allowed a removal efficiency decrease for both Cr(VI) and Congo red. This behavior is attributed to many factors such as the charge in adsorbates and the adsorbent surface charge in the water environment. The increased removal efficiency at very low pH values is attributed to the high electrostatic attractions between the negatively charged adsorbates (acidic CR dye and Cr(VI)) and the protonated adsorbent functional groups. The decreased removal efficiency at a higher pH value is attributed to the existence of negatively charged hydroxyl groups that compete with adsorbate molecules for adsorption active sites. Additionally, Cr_2_O_7_^2−^ and HCrO_4_^−^ are the predominant ions of chromium in the pH range of (2.0 to 6.0), while this form changes by a pH increase to HCrO_4_^2−^. It is well known that the HCrO_4_^−^ form of chromium ions has the lowest adsorption energy equal to (0.60 to 2.50 kcal.mol^−1^) [45] that explains their high removal at a lower pH. Moreover, at a low pH, H^+^ ions neutralize the negatively charged adsorbent surface, thereby reducing the barrier to diffusion of dichromate ions. However, the pH effect may be controlled by the development of an electric double layer over the adsorbent. With the increase in pH, the concentration of H^+^ ions changes from acidic to basic, and, consequently, the polarity of the double layer at the adsorbent surface may be changed from positive to negative. At a lower pH, the system attained equilibrium faster and also the percentage of chromium adsorbed increased, as explained by Verma et al. [46]. Finally, we can conclude that the adsorption behavior at different pH values is dependent on the protonation or deprotonation of the adsorbent functional groups which detect the attraction or repulsion forces between these functional groups and metallic ions and dyes. Therefore, the adsorbent active binding sites and surface chemistry of the material are clearly influenced by the pH, which affects the efficiency of the adsorption.

#### 3.2.4. Initial Concentration Effect

Figure 3d shows the effect of using different Congo red dye and Cr(VI) initial concentrations on their adsorption over the PPy/Fe_3_O_4_/SiO_2_ nanocomposite. This was achieved by using a concentration range of 25.0–300.0 mg.L^−1^ for both Congo red dye and Cr(VI). According to Figure 3d, the adsorption capacity increased by increasing the initial concentration of Congo red dye and Cr(VI), and at a certain point, equilibrium was reached (the adsorption increase became slower). Equilibrium was reached at 100.0 mg.L^−1^ for both Congo red dye and Cr(VI), which is considered the optimum initial concentration. The maximum adsorption capacities reached 207.0 mg.L^−1^ and 257.0 mg.L^−1^ for Cr(VI) and Congo red dye, respectively. These highest values of pollutant removal at higher concentrations could be explained on the basis of the high attraction forces between pollutants and the adsorbent surface at high concentrations.

#### 3.2.5. Adsorption Isotherm

**Figure 4:**
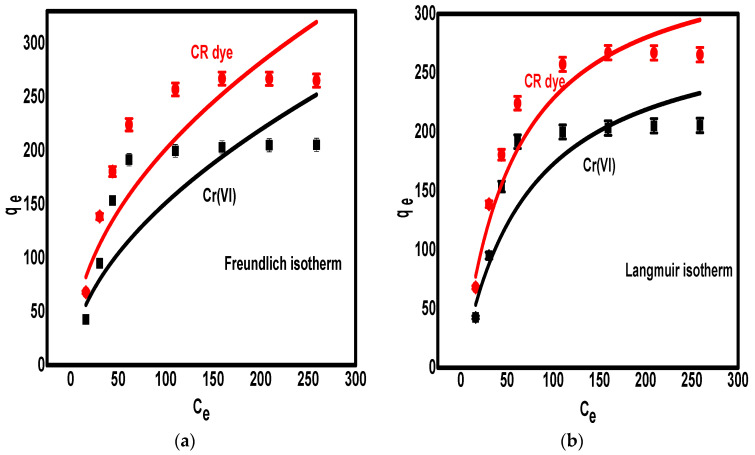

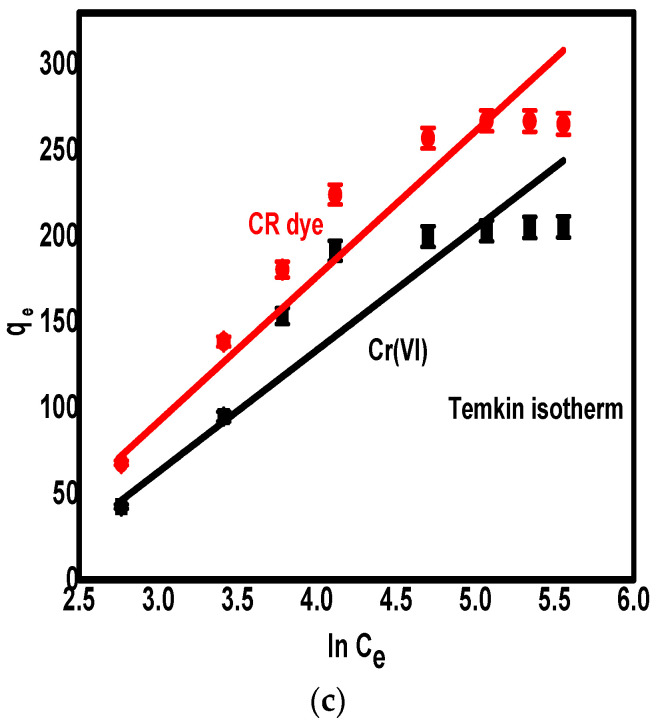
Plotting of Freundlich isotherm (**a**), Langmuir isotherm (**b**), and Temkin isotherm (**c**) for the removal of Congo red dye and Cr(VI) metal ions over the PPy/Fe_3_O_4_/SiO_2_ nanocomposite.

Freundlich, Langmuir, and Temkin adsorption isotherms were used to investigate the adsorption isotherm of Congo red dye and Cr(VI) ions over the surface of the PPy/Fe_3_O_4_/SiO_2_ nanocomposite. The main purpose of studying the adsorption isotherm is to understand the adsorption mechanism over the studied adsorbent (i.e., assess the adsorption limit and the distribution of the pollutants on the adsorbent sites). The fitting of isotherms is presented in Figure 4.

The first studied isotherm was the Freundlich model that suggested heterogeneous adsorption of contaminants over the surface of the adsorbent, and the adsorption was achieved via the construction of multilayers from the pollutant ions [47]. The Freundlich model can be represented by the equation

q_e_ = K_F_ + C_e_^N^, (1)

where q_e_ denotes the amount of uptake pollutants after equilibration (mg.g^−1^), C_e_ denotes the rest concentrations (mg.L^−1^), and N and K_F_ are the normal constants of the Freundlich model and related to the adsorption intensities and capacities, respectively.

The second studied isotherm was the Langmuir model that suggested homogenous adsorption of contaminants over the surface of the adsorbent, and the adsorption was achieved via the construction of a monolayer from the pollutant ions [48]. The Langmuir model can be represented by the equation

q_e_ = K_L_q_m_/(1 + K_L_C_e_), (2)

where K_L_ denotes the Langmuir constant and q_m_ denotes maximum adsorption after reaching saturation.

The last model is the Temkin isotherm that indicates the effect of sorbate ions on each other during the adsorption process. The Temkin model can be represented by the equation

q_e_ = B lnA + B lnC_e_, (3)

where B and A denote Temkin model constants. Table 1 summarizes the Freundlich and Langmuir parameters for the removal of Congo red dye and Cr(VI) ions over the PPy/Fe_3_O_4_/SiO_2_ nanocomposite.

According to the data in Table 1, there was a lower fit of the Freundlich and Temkin isotherms than the Langmuir isotherm for the adsorption of Congo red dye and Cr(VI) metal on the surface of the PPy/Fe_3_O_4_/SiO_2_ nanocomposite, as indicated from the values of R^2^. The maximum adsorption capacities of the PPy/Fe_3_O_4_/SiO_2_ nanocomposite calculated using the Langmuir model were found to be 298.22 mg.g^−1^ and 361.43 mg.g^−1^ for Cr(VI) and Congo red dye, respectively. Subsequently, the adsorption of Cr(VI) and Congo red dye on the surface of the PPy/Fe_3_O_4_/SiO_2_ nanocomposite was achieved in the form of monolayer uptake on the energetically symmetrical (homogeneous) adsorption sites. Additionally, the values of N obtained from fitting of the experimental data with the Freundlich isotherm were higher than zero, indicating the favorable adsorption of both pollutants. Additionally, the experimental data also fitted the Temkin model, indicating that the adsorption process was possibly affected by pollutant ion interactions.

#### 3.2.6. The Regeneration Study

**Figure 5:**
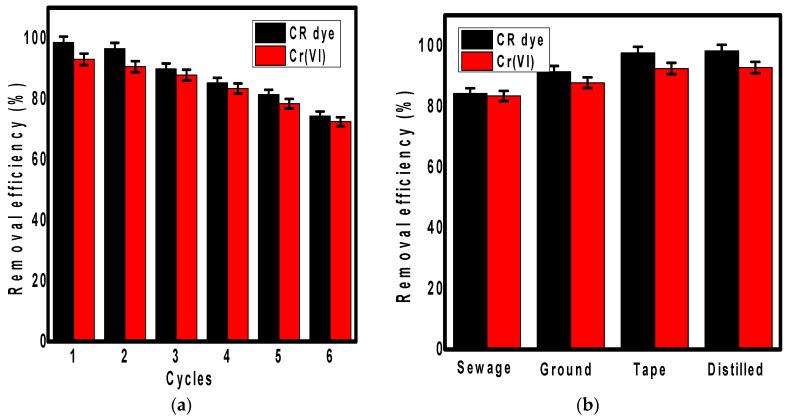
The regeneration study of PPy/Fe_3_O_4_/SiO_2_ nanocomposite for the removal of Congo red dye and Cr(VI) up to six successive cycles (**a**) and the effect of water type (**b**).

The application of any studied adsorbent at a large scale for wastewater treatment essentially requires an investigation of its regeneration [49,50]. Concerning this, the reusability of the PPy/Fe_3_O_4_/SiO_2_ nanocomposite for the removal of Cr(VI) ions and Congo red dye was studied for up to six successive cycles, as shown in Figure 5a.

After each cycle, the adsorbent was separated using an external magnet, washed several times extensively with distilled water to remove any adhered pollutants, and then dried in an oven to be used in the next adsorption–desorption cycle. The studied PPy/Fe_3_O_4_/SiO_2_ nanocomposite showed good reusability for the removal of Cr(VI) and Congo red dye during the studied cycles, with a decrease in the efficiencies by increasing the cycles. The composite’s efficiency to remove Congo red dropped from 98.0% to 74.0%, while for Cr(VI) removal, it dropped from 93.0% to 72.0% after six reuse cycles. This behavior is attributed to the destroyed adsorption sites over the nanocomposite surface after each cycle, causing the decrease in the material efficiency to capture the pollutants.

#### 3.2.7. The Water Type Effect

The removal efficiency of Cr(VI) ions and Congo red dye on the surface of the PPy/Fe_3_O_4_/SiO_2_ nanocomposite using different types of water including sewage water, groundwater, tap water, and distilled water was investigated to determine the applicability of the synthesized nanocomposite for an effective real water treatment (Figure 5b). The order of the removal efficiency for both pollutants (Cr(VI) and Congo red dye) was distilled water > tap water > groundwater > sewage water. This resulting behavior is attributed to the existence of other contaminants in each water type that compete with the studied pollutants for the adsorption active sites over the nanocomposite surface. Furthermore, the selectivity of any innovative adsorbent should be studied toward different competing co-ions including bicarbonate, sulfate, and chloride ions that may be present in water. For example, it was reported that sulphate and chloride negatively affect the adsorption of Cr(VI) on polypyrrole@magnetic chitosan nanocomposites [51]. Additionally, the similarity of the ionic charge between Cr(VI) and bicarbonate ions showed a competitive effect on the adsorption of Cr(VI) [51,52]. Therefore, it is very important to determine the chemical composition of the water before starting the adsorption experiments. Matched to the reported concentrations in nature, these removal efficiencies were achieved for a pollutant concentration of 100.0 mg.L^−1^, which is considered high. This fact approves the applicability of the PPy/Fe_3_O_4_/SiO_2_ nanocomposite for real water treatment.

#### 3.2.8. Comparative Study

The assessment of the synthesized nanocomposite for the removal of Congo red dye and Cr(VI) ions required its comparison with previously reported adsorbents. In this regard, we summarized the previously studied adsorbents for the removal of Congo red dye and Cr(VI) ions in Table 2. According to Table 2 [53,54,55,56,57,58,59,60,61,62,63,64,65,66,67,68], it is noticeable that the present synthesized PPy/Fe_3_O_4_/SiO_2_ nanocomposite showed a good removal capacity for the removal of Cr(VI) ions and Congo red dye when compared to previously reported materials. However, the difference in the adsorbent efficiency between materials may be controlled by the variability of the interactions between pollutants and the material functional groups. Strong interactions with specific functional groups present on the adsorbent surface allow a high adsorption capacity [67]. Furthermore, the competitive potential application of these innovative adsorbents should be evaluated taking into consideration different parameters associated with the material characteristics (regeneration, degradation, life cycle, etc.) and with the wastes resulting from the process (loaded pollutant disposal, chemicals used for the adsorption–desorption, etc.). Moreover, a cost comparison between these materials should be conducted.

## 4. Conclusions

In this study, a promising polymer-based nanocomposite, PPy/Fe_3_O_4_/SiO_2_, was synthesized for the elimination of Congo red dye and Cr(VI) from water. The synthesized nanocomposite was characterized using different techniques (XRD, FT-IR, SEM, etc.). XRD results confirmed the crystalline structure of Fe_3_O_4_ nanoparticles that decreased by the addition of PPy and silica. Additionally, SEM images confirmed the size of the synthesized materials. FT-IR bands approved the successful combination between three components of the hybrid Fe_3_O_4_, SiO_2_, and PPy. The synthesized nanocomposite was examined for the removal of Cr(VI) and Congo red dye from water. The effects of different factors including the contact time, adsorbent dosage, solution pH, and initial concentration on the adsorption process were studied to detect the optimum conditions. The adsorption data were found to be more fitting to the Langmuir model with maximum adsorption of 298.22 mg.g^−1^ and 361.43 mg.g^−1^ for the removal of Cr(VI) and Congo red, respectively. This indicated the monolayer adsorption of Cr(VI) and Congo red on the energetically homogeneous active sites of the PPy/Fe_3_O_4_/SiO_2_ nanocomposite. The regeneration study indicated the ability to reuse the nanocomposite several times for the removal of Cr(VI) and Congo red, which may reduce the overall cost of the treatment. Finally, we can conclude that the PPy/Fe_3_O_4_/SiO_2_ nanocomposite is a promising material for water treatment and must be examined in the future for additional pollutant removal.

## Figures and Tables

**Table 1:** The calculated Langmuir and Freundlich isotherm parameters for the removal of Cr(VI) and CR dye over PPy/Fe_3_O_4_/SiO_2_ nanocomposite.

Pollutant	Langmuir				Freundlich		Temkin		
	q_max_ (mg.g^−1^)	K_L_ (L.mg.g^−1^)	R^2^	N	K_F_ (L.mg.g^−1^)	R^2^	B (J.mol^−1^)	A (L.g^−1^)	R^2^
CR dye	361.43	0.017	0.946	0.487	2.1360	0.842	69.44	4.33	0.931
Cr(VI)	298.22	0.013	0.892	0.537	1.2720	0.785	57.12	4.52	0.835

**Table 2:** Comparative assessment of CR dye and Cr(VI) ions adsorption capacity of PPy/Fe_3_O_4_/SiO_2_ nanocomposite with previously reported adsorbents.

Adsorbent	Pollutant	q_m_ (mg.g^−1^)	Ref.
PPy/Fe_3_O_4_/SiO_2_	CR dye and Cr(VI)	361.43 and 298.22	This study
PPy/Fe_3_O_4_/AgCl	Cr(VI)	111	[52]
PPy-rGO/Fe_3_O_4_	Cr(VI)	227	[53]
PPy-coated halloysite nanotubes	Cr(VI)	149	[54]
PPy-PANI fibers	Cr(VI)	227	[55]
Glycine-doped PPy	Cr(VI)	217	[56]
PPy/Fe_3_O_4_	Cr(VI)	169	[57]
Fe_3_O_4_ glycine-doped PPy	Cr(VI)	238	[58]
Aspartic acid-doped PPy	Cr(VI)	177	[59]
Hierarchical porous MgBO_2_(OH) microspheres	CR dye	228	[60]
Mesoporous activated carbon	CR dye	189	[61]
NiO nanosheets	CR dye	168	[62]
MgO powders	CR dye	105	[63]
Neem leaf powder2	CR dye	41	[64]
Magnetic core–manganese oxide shell	CR dye	42	[65]
Chitosan/montmorillonite nanocomposite	CR dye	55	[66]
Ashitaba waste-based activated carbons	CR dye	289–381	[67]
Walnut shell-based activated carbons	CR dye	314–400	[68]
Nanofibrous membranes from ion polymers	CR dye	70.8	[69]

## Data Availability

Data sharing is not applicable.
